# Cross-Talk between *Staphylococcus aureus* and Other Staphylococcal Species via the *agr* Quorum Sensing System

**DOI:** 10.3389/fmicb.2016.01733

**Published:** 2016-11-08

**Authors:** Jaime Canovas, Mara Baldry, Martin S. Bojer, Paal S. Andersen, Bengt H. Gless, Piotr K. Grzeskowiak, Marc Stegger, Peter Damborg, Christian A. Olsen, Hanne Ingmer

**Affiliations:** ^1^Department of Veterinary Disease Biology, Faculty of Health and Medical Sciences, University of Copenhagen Frederiksberg, Denmark; ^2^Department of Microbiology and Infection Control, Statens Serum Institut Copenhagen, Denmark; ^3^Center for Biopharmaceuticals and Department of Drug Design and Pharmacology, Faculty of Health and Medical Sciences, University of Copenhagen Copenhagen, Denmark

**Keywords:** *Staphylococcus aureus*, *Staphylococcus schleiferi*, quorum sensing, *agr*, quorum sensing inhibition, auto-inducing peptide, cross-talk, anti-virulence therapy

## Abstract

Staphylococci are associated with both humans and animals. While most are non-pathogenic colonizers, *Staphylococcus aureus* is an opportunistic pathogen capable of causing severe infections. *S. aureus* virulence is controlled by the *agr* quorum sensing system responding to secreted auto-inducing peptides (AIPs) sensed by AgrC, a two component histidine kinase. *agr* loci are found also in other staphylococcal species and for *Staphylococcus epidermidis*, the encoded AIP represses expression of *agr* regulated virulence genes in *S. aureus*. In this study we aimed to better understand the interaction between staphylococci and *S. aureus*, and show that this interaction may eventually lead to the identification of new anti-virulence candidates to target *S. aureus* infections. Here we show that culture supernatants of 37 out of 52 staphylococcal isolates representing 17 different species inhibit *S. aureus agr*. The dog pathogen, *Staphylococcus schleiferi*, expressed the most potent inhibitory activity and was active against all four *agr* classes found in *S. aureus*. By employing a *S. aureus* strain encoding a constitutively active AIP receptor we show that the activity is mediated via *agr*. Subsequent cloning and heterologous expression of the *S. schleiferi* AIP in *S. aureus* demonstrated that this molecule was likely responsible for the inhibitory activity, and further proof was provided when pure synthetic *S. schleiferi* AIP was able to completely abolish *agr* induction of an *S. aureus* reporter strain. To assess impact on *S. aureus* virulence, we co-inoculated *S. aureus* and *S. schleiferi in vivo* in the *Galleria mellonella* wax moth larva, and found that expression of key *S. aureus* virulence factors was abrogated. Our data show that the *S. aureus agr* locus is highly responsive to other staphylococcal species suggesting that *agr* is an inter-species communication system. Based on these results we speculate that interactions between *S. aureus* and other colonizing staphylococci will significantly influence the ability of *S. aureus* to cause infection, and we propose that other staphylococci are potential sources of compounds that can be applied as anti-virulence therapy for combating *S. aureus* infections.

## Introduction

At least 40 different *Staphylococcus* species have been described to date (Harris et al., 2002). While a large number of these are found to colonize humans, many also colonize animals (Nagase et al., 2002). *Staphylococcus aureus* is by far the best characterized staphylococcal species. This opportunistic pathogen resides on skin and mucosal membranes in humans and animals, and it can cause a variety of infections ranging from mild skin and soft tissue infections to severe conditions such as septicemia (Lowy, 1998). Expression of the majority of *S. aureus* virulence factors is controlled by the accessory gene regulator (*agr)* quorum sensing system. *agr* is composed of two divergent transcripts: one (P2) encoding the AgrAC two component signal transduction system that responds to autoinducing peptides (AIPs) encoded and secreted by the products of *agrBD*; and the other (P3) encoding a regulatory RNA, RNAIII, the effector molecule of *agr*. AIPs bind to the *agrC*-encoded histidine kinase (AgrC), and via phosphorylation of the AgrA response-regulator, stimulate the expression of RNAIII (Wang et al., 2014). At high cell densities, AIP accumulation results in up-regulation of exoprotein expression including the *hla*-encoded virulence factor α-hemolysin, and down-regulation of surface-associated proteins such as *spa*-encoded protein A (Queck et al., 2008; Shoham, 2011).

*Staphylococcus aureus* strains can be divided into at least four *agr* classes (groups I–IV), with distinct AIPs inducing virulence gene expression within each group, but repressing expression in strains of other groups by acting as competitive inhibitors to AgrC binding (Ji, 1997; Otto et al., 2001; Geisinger et al., 2012). The *agr* specificity is determined by AgrB, AgrC, and AgrD, which vary among the four groups. *agr* has also been identified in other staphylococcal species although with much higher sequence divergence. Despite this diversity, functional *agr* loci have been demonstrated in *Staphylococcus lugdunensis* and *Staphylococcus epidermidis* (Vandenesch et al., 1993; Wamel et al., 1998). *S. epidermidis* is another clinically important opportunistic pathogen whose virulence is largely controlled via the *agr* system (Olson et al., 2014), and the *S. epidermidis* AIP is a potent inhibitor of the *S. aureus agr* system (Otto et al., 2001). Although, the biological importance of this cross-inhibition of quorum sensing remains unknown, it has been suggested that it contributes to niche competition between *S. epidermidis* and *S. aureus*, resulting in the predominance of *S. epidermidis* on the skin and in indwelling device-associated chronic infections (Otto et al., 2001; Thoendel et al., 2011). The frequent isolation of other staphylococci together with *S. aureus* also point to a possible interaction between staphylococcal species with a role in niche competition and colonization (Lina et al., 2003; Kozioł-Montewka et al., 2006).

The aim of this study was to examine the extent to which *agr* cross-inhibitory activity occurs between *S. aureus* and other staphylococcal species, and to elucidate the mechanism behind this cross-talk. Understanding this interaction between staphylococci may help in the identification of new anti-virulence strategies targeting *S. aureus* infections.

## Materials and Methods

### Bacterial Strains and Growth Conditions

*Staphylococcus aureus* strains used in this study include: Strain 8325-4 (Novick, 1967) was used as a source of AIP-I containing supernatant. For the β-galactosidase plate assays PC203 (*S. aureus* 8325-4, *spa::lacZ*), PC322 (*S. aureus* 8325-4, *hla::lacZ*), SH101F7 (*S. aureus* 8325-4, *rnaIII::lacZ*) (Chan and Foster, 1998) and strain MOZ53 *S. aureus agr* type III (Horsburgh et al., 2002) were used. For the β-lactamase liquid assay strain RN10829 WT and RN10829 Const (Geisinger et al., 2009) a β-lactamase reporter strain substituting the native *agr* locus with a chromosomal integration of P2-*agrA* and P3-*blaZ* and a plasmid from which a constitutive active variant of AgrC (*agrC-I-R238H*) is expressed, was used to assess AgrC-dependent effects of staphylococcal supernatants. Fluorescent reporters AH1677, AH430, AH1747, and AH1872 (Hall et al., 2013) were used to evaluate *agr* induction of the four different *S. aureus agr* groups. We cloned the *Staphylococcus schleiferi agrBD* genes into the *BglII/EcoRI* sites of expression vector pRAB12-lacZ (Helle et al., 2011) using primers 5′-GATACAAGATCTGTTAAGGAGGAGGGCTATTTG-3′ and 5′-GATACAGAATTCCGCTCTCTAAACATTATTTTATTATTC-3′ and chromosomal DNA from *S. schleiferi* strain 2898 as template generating plasmid pRAB12-agrBD_Ss_. This construct or the vector itself was expressed in the *S. aureus agr* deletion strain 8325-4Δ*agr* [constructed by transduction (φ11) from strain RN6911 (Novick et al., 1993) into strain 8325-4] by growing the strains overnight under induction (0.2 μg/ml anhydrotetracycline) generating AIP_Ss_ containing or AIP negative supernatants respectively. All other staphylococcal strains used are listed in **Table 1**. Unless otherwise stated, bacteria were grown in Tryptone Soya Broth (TSB), Oxoid (1:10 volume/flask ratio), at 37°C with shaking at 200 rpm.

**Table 1:** Staphylococcal strains, their origin, and RNAIII-regulation activity.

Id.	Name	Host isolation source	*rnaIII* downregulation	Id.	Name	Host isolation source	*rnaIII* downregulation
27472	*Staphylococcus intermedius*	Dog wound	++	450	*Staphylococcus equorum*	Horse nose	X
30755	*Staphylococcus pseudintermedius*	Dog skin	++	1312	*Staphylococcus haemolyticus*	Dog ear	+
C-31106	*Staphylococcus pseudintermedius*	Dog wound	++	2181	*Staphylococcus haemolyticus*	Bird trachea	X
C- 31304	*Staphylococcus pseudintermedius*	Dog nose	++	28993	*Staphylococcus haemolyticus*	Bird trachea	+
30510	*Staphylococcus pseudintermedius*	Dog urine	+	9525	*Staphylococcus hominis*	Not reported	+
30665	*Staphylococcus pseudintermedius*	Dog skin	++	1084	*Staphylococcus hominis*	Cat urine	+
30703	*Staphylococcus pseudintermedius*	Dog wound	++	218	*Staphylococcus hyicus*	Pig joint	+ + ++
30106	*Staphylococcus warneri*	Dog wound	+	2808	*Staphylococcus hyicus*	Not reported	+ + ++
29927	*Staphylococcus warneri*	Dog urine	+	4948	*Staphylococcus hyicus*	Not reported	+ + ++
30331	*Staphylococcus warneri*	Dog skin	+	6948	*Staphylococcus lentus*	Mink skin	X
28351	*Staphylococcus xylosus*	Cow ear	X	6949	*Staphylococcus lentus*	Mink Skin	X
L136	*Staphylococcus xylosus*	Horse abscess	X	C-30966-5^a^	*Staphylococcus lentus*	Mink skin	X
9529	*Staphylococcus capitis*	Not recorded	++	62	*Staphylococcus vitulinus*	Horse trachea	+
2877	*Staphylococcus capitis*	Dog ear	X	96	*Staphylococcus vitulinus*	Horse wound	+
6994	*Staphylococcus capitis*	Dog tonsil	X	128	*Staphylococcus vitulinus*	Horse wound	+
52	*Staphylococcus capitis*	Horse uterus	++	30689-20	*Staphylococcus schleiferi*	Mink skin	+ + ++
53	*Staphylococcus chromogenes*	Horse uterus	+++	30743	*Staphylococcus schleiferi*	Dog ear	+ + ++
2890	*Staphylococcus chromogenes*	Cow wound	++	30219	*Staphylococcus schleiferi*	Dog skin	+ + ++
313	*Staphylococcus chromogenes*	Cow milk	++	2319	*Staphylococcus schleiferi*	Cat	++
1069	*Staphylococcus epidermidis*	Dog urine	X	2862	*Staphylococcus schleiferi*	Dog ear	++
352	*Staphylococcus epidermidis*	Ox milk	X	2898	*Staphylococcus schleiferi*	Mink skin	+ + ++
389	*Staphylococcus epidermidis*	Not recorded	+	9468	*Staphylococcus sciuri*	Not reported	X
30575	*Staphylococcus epidermidis*	Dog kindney	X	9470	*Staphylococcus sciuri*	Not reported	X
29886	*Staphylococcus delphini*	Horse wound	+ + +	9505	*Staphylococcus sciuri*	Not reported	+
C-30966-8^a^	*Staphylococcus delphini*	Mink skin	+ + +	1457	*Staphylococcus simulans*	Dog	+ + +
C-31232-2	*Staphylococcus delphini*	Mink wound	X	312	*Staphylococcus simulans*	Ox milk	+ + +

*Down-regulation was rated according to the size of the inhibition halo around the well where: X: no effect; + slight effect; ++: moderate effect; +++: severe effect and ++++: very severe effect.*

### β-Galactosidase Plate Assay

The reporter assay was conducted as described by Nielsen et al. (2010). Test supernatants, and control supernatants of strains 8325-4 (AIP-I) and M0Z53 (AIP-III) were used. Incubation until blue color appeared in plates varied from 9 to 48 h.

### *In vitro* Competition As Assessed Using the β-Galactosidase Liquid Assay

The assay was based on the liquid β-galactosidase assay (Miller, 1972) with some modifications. Briefly, Overnight (ON) cultures of our SH101F7 *S. aureus* RNAIII reporter strain and our *S. schleiferi* strains (grown at 37°C while shaking at 200 rpm) were diluted 100x in 15 mL TSB and allowed to reach an OD_600_ of 0.5 and after adjusting each strain to an OD_600_ of 0.1 in TSB the competition was started at a ratio of 1:1 and followed over time. From each culture, 1 mL was taken at each time interval and the samples were sonicated for 10 s to disrupt aggregates formed. The OD_600_ was measured and serial dilutions for each sample were made and plated on TSA with X-gal (150 μg/mL). The remaining samples were centrifuged for 3 min at 8000 rpm and 4°C. After centrifugation, the supernatants were removed and the pellets resuspended in 1 mL of TRIS 50 mM, pH 8 and 3 μL of lysostaphin. The mix was incubated at 37°C for 30 min to allow for cell lysis. Z-buffer (400 μL) was then added to each sample which were further incubated for 5 min at 28°C. Lastly, 100 μL of ONPG (*ortho*-Nitrophenyl-β-galactoside) (4 mg/mL) was added to the mix and the time necessary for the solution to turn yellow was controlled. The OD at 420 and 550 nm for each sample was measured. The activity of the samples was calculated in Miller units using the following formula as described by Miller (1972):

$$Miller\,\;Units\,\;:\frac{1000*\left(OD420-\left(1.75*OD550\right)\right)}{OD600*T*V}$$

Where: T = time of reaction in min;

V = ml cells added to the assay tubes.

### β-Lactamase Assay and Inhibitory Concentration (IC_50_)

The method used is described by Nielsen et al. (2014). Briefly the RN10829 (P2-agrA:P3-blaZ)/pagrC-I (WT) and RN10829(P2-agrA:P3-blaZ)/pagrC-I-R23H (AgrC const.) reporter strains were grown to an OD_600_ of 0.4–0.5 where a 1/10 volume of AIP-I containing supernatant (obtained from strain 8325-4) and 1/10 *S. schleiferi* supernatants were added to the reporter strain culture. In assays using heterologously expressed AIP_Ss_ 1/20 volume of AIP-I containing supernatant was challenged with 1/5 volume supernatant from expression cultures. Samples obtained at 30 min time intervals after addition of test solutions were analyzed for β-lactamase activity by nitrocefin conversion. The IC_50_ of the selected *S. schleiferi* supernatants was also tested using the β-lactamase assay, where a 1/10 volume (0.5 mL) of supernatant was added to the total volume of 5 mL of the reporter strain culture (RN10829-WT) representing the undiluted supernatant (100%). Then, 80, 60, 40, 20, 10, 5, 2.5, and 2% of the initial volume of the selected supernatant was added to obtain the IC_50_ curve.

### Assessment of *agr* Inhibition Across *agr* Groups

Fluorescent *S. aureus* reporter strains of agr types I-IV (P3-*yfp*) were used to evaluate the inhibitory potential of staphylococcal supernatants on respective *agr* types. Individual reporter strains were grown to early exponential phase (non-fluorescent, OD approximately 0.01), then added 20% *S. schleiferi* 2898 stationary phase supernatant or TSB as a control and grown to late stationary phase to allow for full *agr* induction. P3 promoter activity of the bacterial population was monitored as accumulated YFP by flow cytometry on a BD Accuri^TM^ C6 flow cytometer using the FL1 channel.

### Reverse Transcriptase-Quantitiative PCR

Overnight culture of the strain 8325-4 was diluted 100x in 15 mL of TSB in a 300 mL flask. Three replicate cultures were prepared and once the cultures reached an OD_600_ of 0.35 they were split into two different conditions; one with 10% 8325-4 supernatant alone was added and the other with an additional 10% of *S. schleiferi* 2898 supernatant. Cultures were allowed to grow until sample withdrawal at 30 and 60 min. Samples were centrifuged and the pellet was immediately frozen at -80°C. The disruption of the cell membranes was performed with FastPrep and the the QIAGEN RNeasy kit was used to purify the RNA as per manufacturer’s instructions. Genomic DNA was then removed from the samples using DNase-I, RNase-free from Fermentas. The samples were first incubated for 60 min at 37°C, followed by 10 min incubation with 50 mM of EDTA at 65°C. To generate cDNA from the RNA samples, the High Capacity cDNA RT kit from Applied Biosystems was used. 10 μL of RNA and 10 μL of RT-master mix were added to each reaction. The master mix contained RT Buffer, RT random primers, dNTP mix, Nuclease-free water, and reverse transcriptase. The negative controls consisted of samples with no reverse transcriptase added. The samples were run for 10 min at 25°C, 120 min at 37°C, 5 min at 85°C in a standard PCR machine. Finally, 1.5 μL of cDNA was assayed by real-time PCR on a LightCycler^®^ 96 Instrument using FastStart Essential DNA Green Master FastStart Essential DNA Green Master (Roche) and primers for the reference genes (*ileS, pyk*) and the target gene (*rnaIII*) as presented in **Table 2**.

**Table 2:** Real time PCR primers used in this study for the assessment of reference (*ileS, pyk*) and target gene (*rnaIII*) expression.

Gene	Sequence forward	Sequence reverse
*rnaIII*	GCACTGAGTCCAAGGAAACTAAC	AAGCCATCCCAACTTAATAACC
*ileS*	ACATACAGCACCAGGTCACG	CGCCTTCTTCAGTAAATACACC
*pyk*	AGGTTGAACTCCCCAAACAA	GCAGCCCAAGATTACAAAAA

### *In vivo* Competition Assay with *Galleria mellonella*

The *Galleria mellonella* infection was carried out as described by Pollitt et al. (2014) with minor modifications. Briefly, fifth-instar *G. mellonella* larvae were inoculated (in a proleg) with a total of 2 × 10^7^ CFU/mL of *S. aureus* (SH101F7 *rnaIII*::*lacZ*), *S. schleiferi* (2898 erythromycin resistant) or a co-culture of the two strains (to a combined final CFU/mL of 2 × 10^7^) and split into groups for CFU counting (35 per group) and survival benefit observation (20 per group). Two control groups were included; one injected with phosphate buffered saline (PBS) and the other not handled at all. The larvae were then incubated at 37°C. At 24, 48, and 72 h post-inoculation, the hemolymph of the larvae was collected for CFU determination. Colonies were counted after O/N incubation at 37°C on TSA containing erythromycin (5 μg/mL) and X-gal (150 μg/mL). Survival of the larvae was monitored at the same time points as hemolymph collection. This experiment was repeated three times using different batches of larva purchased from a local pet store (HPReptiles, Copenhagen) with similar results.

### AIP Sequencing

Several *Staphylococcus* spp. were sequenced to look for the *agrD* and the amino acid sequence of the AIP. DNA from the selected staphylococci isolates was extracted using the DNeasy Blood and Tissue kit as described by the manufacturer (Qiagen, Valencia, CA, USA). For sequencing preparation fragment libraries were constructed using the Nextera kit (Illumina, San Diego, CA, USA) followed by 251-bp paired-end sequencing on a MiSeq sequencer (Illumina) according to the manufacturer’s instructions. The sequencing reads were assembled using CLC Genomics Work- bench 8.0 (Qiagen, Aarhus, Denmark) with default parameters to include only contigs of 500 nucleotides and with over 30-fold average coverage. The sequence of *S. schleiferi* 2898 has been deposited in GenBank (accession number PRJEB15874). All other sequence contigs are available upon request. Extracted AIP sequences were aligned using Muscle as implemented in MEGAv 6.06, where the phylogeny was constructed using the maximum parsimony approach with 100 bootstraps and represented with midpoint rooting.

### Chemical Synthesis of AIP

The AIP was synthesized by adopting a protocol based on linear peptide hydrazides, which was previously reported by Liu and coworkers (Zheng et al., 2013) The linear peptide hydrazide was synthesized by standard 9-fluorenylmethyloxycarbonyl (Fmoc) solid-phase peptide synthesis (SPPS) using HCTU/*i*-Pr_2_EtN for amino acid activation on an automated peptide synthesizer (SyroWave XP, Biotage). A hydrazide-2-chlorotrityl resin was applied to afford the desired C-terminal peptide hydrazide [YPFCIAYF-NHNH_2_] after nine coupling/deprotection cycles, followed by concomitant deprotection of side chain functionalities and cleavage from the solid support with CF_3_COOH-*i*-Pr_3_SiH-water (95:2.5:2.5, 5 mL, 3 h, room temperature). The crude peptide was obtained by trituration with cold ether and used without any further purification. ESI-MS m/z calcd for C_53_H_68_N_10_O_10_S 1037.5, found 1037.2 [M+H^+^].

The crude linear peptide (10 mg, 0.01 mmol) in DMF (20 μL) was added to a solution of NaNO_2_ (6 mg, 0.09 mmol) in sodium phosphate buffer (0.1 mM, pH 3.0) containing guanidinium chloride (6 M) at -20°C. The reaction mixture was kept at -20°C for 20 min and was then quenched by addition of DMF (0.2 mL) and cyclization buffer [sodium phosphate buffer (0.6 mL, 0.1 mM, pH 6.5) containing thiophenol (44 mg, 0.4 mmol)]. This reaction mixture was agitated on a rocking table for 16 h at room temperature. The desired AIP was then purified by preparative reversed-phase HPLC on a C18 Phenomenex Luna column (250 mm × 20 mm, 5 μm, 100 Å) using an Agilent 1260 LC system equipped with a diode array UV detector, applying a gradient of eluent I (water-MeCN-TFA, 95:5:0.1) and eluent II (0.1% TFA in acetonitrile) with a flow rate of 20 mL/min. Lyophilization of the fractions containing product, provided a white fluffy solid (∼0.5 mg, 5%) at >98% homogeneity as determined by UPLC–MS analysis at 254 nm. The compound was reconstituted in DMSO and accurate concentration was determined by UV spectroscopy (5 mM) before use. ESI-MS m/z calcd for C_53_H_64_N_8_O_10_S 1005.5, found 1005.2 [M+H^+^]. MALDI-TOF MS found 1005.6 [M+H^+^].

### Statistics

Where applicable for the β-lactamase assays (**Figures 2B** and **4A**) statistical analysis was performed using the multiple *t*-test allowing unequal variance. For the qPCR data (**Figure 2A**) an unpaired *t*-test was performed on normalized and log transformed data. For survival curve analysis, the Kaplan–Meier method was applied and statistical analysis was carried out using the Log Rank (Mantel–Cox) Chi square test. Any value above *P* = 0.05 was considered as not significant. All statistical tests were performed with GraphPad Prism v. 7.0.

## Results

### **S. aureus** Virulence Gene Expression is Modulated by Staphylococcal Culture Supernatants

**FIGURE 1:**
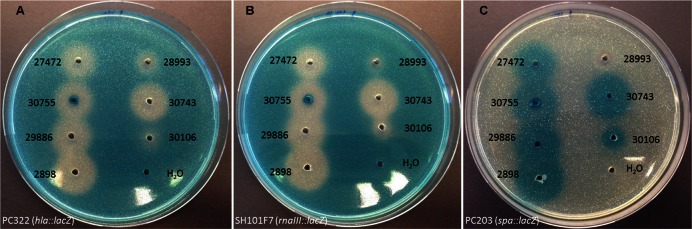
**Modulation of *Staphylococcus aureus* virulence gene expression by staphylococcal culture supernatants.** TSA agar plates (with erythromycin and X-gal) containing **(A)** the *hla-lacZ* (PC322; Ery^r^), **(B)** the *rnaIII-lacZ* (SH101F7; Ery^r^), or **(C)** the *spa-lacZ* (PC203; Ery^r^) reporter strain of *S. aureus* were exposed to 20 μL (in pre-drilled wells) of supernatants from centrifugation (8000 rpm for 60 s) of overnight cultures of strains 27472 (*Staphylococcus intermedius*), 28993 (*Staphylococcus haemolyticus*), 30755 (*Staphylococcus pseudintermedius*), 30743 (*Staphylococcus schleiferi*), 29886 (*Staphylococcus delphini*), 30106 (*Staphylococcus warneri*) and 2898 (*Staphylococcus schleiferi*). H_2_O was used as a control. Zones appeared between 9 and 36 h of incubation at 37°C. This figure is representative of one set of screening plates.

A total of 52 staphylococcal isolates representing 17 species obtained from a variety of different animal hosts (**Table 1**) were examined for their ability to interfere with the *agr* quorum sensing system of *S. aureus* using a previously established reporter assay (Nielsen et al., 2010). Staphylococcal strains to be tested were grown overnight to stationary phase in tryptone soy broth (TSB), and cell-free supernatants were added to wells formed in tryptone soy agar (TSA) plates containing reporter strains carrying *lacZ* fusions to either one of the *agr* controlled virulence genes *hla*, *spa*, or *rnaIII* as well as the β-galactosidase substrate, 5-bromo-4-chloro-3-indolyl-β-D-galactopyranoside (X-Gal). For the majority (37 out of 52) of the staphylococcal supernatants tested we observed a reduced expression of *hla* and *rnaIII* but increased *spa* expression (**Figure 1**; **Table 1**). The 37 supernatents exhibiting this expression pattern represents 14 of the 17 staphylococcal species, indicating that these secrete substances that interfere with the *agr* system of *S. aureus*.

### The Dog Pathogen *Staphylococcus schleiferi* is a Potent Inhibitor of the *S. aureus agr* Quorum Sensing System

**FIGURE 2:**
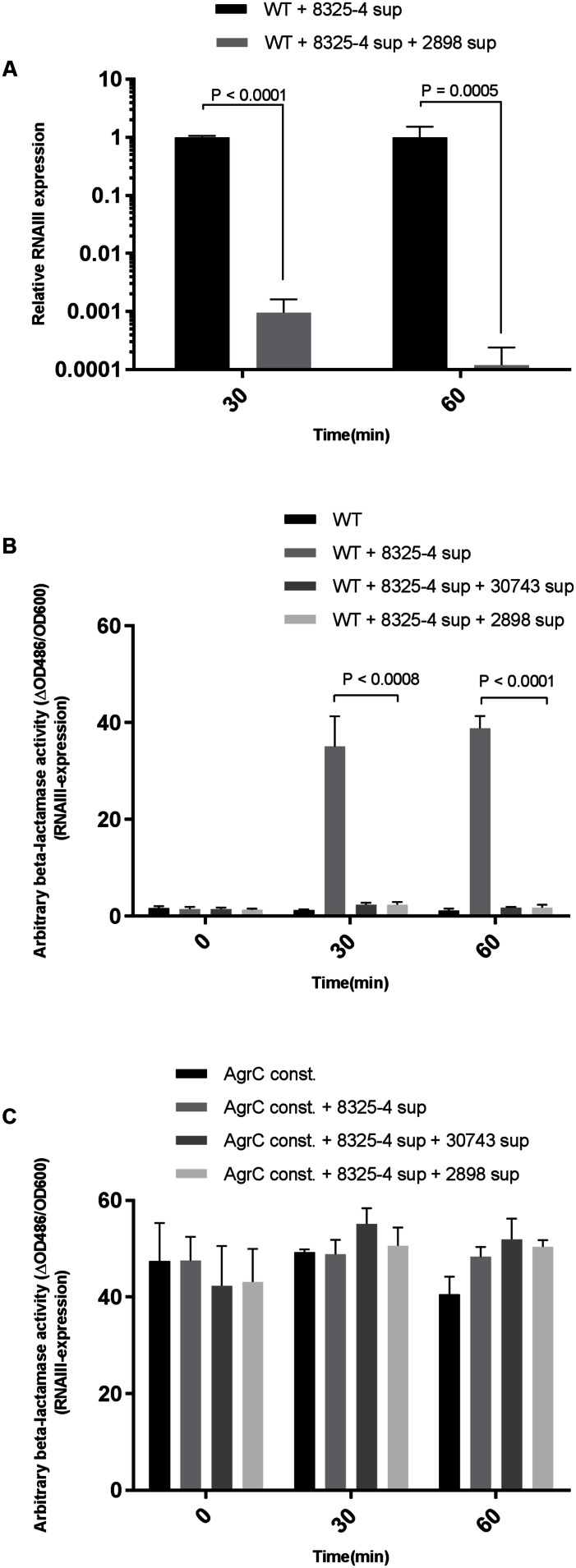
**Quantification of interference by *S. schleiferi* on RNAIII expression in *S. aureus.* (A)** RT-qPCR quantitative verification of RNAIII downregulation in the *S. aureus* 8325-4 strain. *S. aureus* in 15 mL TSB was grown to an OD_600_ = 0.35 and supplemented with a 1:10 volume of *S. schleiferi* 2898 supernatant. Samples were collected 30 and 60 min post-exposure to *S. schleiferi* supernatant, RNA was isolated, cDNA prepared (and RNAIII levels were quantified. Interference of *S. schleiferi* supernatant on *S. aureus agr* was monitored using the strains **(B)** RN10829(P2-agrA:P3-blaZ)/pagrC-I (WT) and **(C)** RN10829(P2-agrA:P3-blaZ)/pagrC-I-R23H (AgrC const.) Reporter strains were grown to an OD_600_ of 0.4–0.5 where a 1/10 volume of AIP-I containing supernatant from strain 8325-4 and 1/10 *S. schleiferi* supernatant were added to the reporter strain culture. Samples obtained at 30 min time intervals after addition of test solutions were analyzed for β-lactamase activity by nitrocefin conversion (Nielsen et al., 2014). Each bar represents the average of 3 biological replicates and the error bars represent the standard deviation.)

The magnitude of *agr*-interference caused by staphylococcal supernatants was monitored in *S. aureus* 8325-4 by RT-qPCR using previously described primers (Nielsen et al., 2012). Using the supernatant of the notably active *S. schleiferi* strain 2898 we observed that the relative RNAIII expression in *S. aureus* 8325-4 decreased by 300 and 3000-fold at 30 and 60 min respectively, when compared to unexposed cells (**Figure 2A**). Furthermore, the 50% inhibitory concentration (IC_50_) was reached with *S. schleiferi* supernatant constituting only 6% of the total *S. aureus* culture volume.

To address if the observed effect of staphylococcal supernatants on virulence gene expression in *S. aureus* is mediated via direct interference with the *S. aureus agr* regulatory system, we examined if the effect could be mitigated in a *S. aureus* strain encoding a constitutively active AgrC sensor histidine kinase (Geisinger et al., 2009). Using the reporter strains RN10829 WT (expressing the WT AgrC) and RN10829 Const. (isogenic mutant expressing the constitutively active AgrC) containing a *blaZ* gene fused to the P3 promoter as previously described by Nielsen et al. (2014), we examined the culture supernatants obtained from *S. schleiferi* strains 2898 and 30743. As predicted, in the presence of the WT AgrC in the reporter strain, supernatants of both of the *S. schleiferi* strains significantly down-regulated RNAIII expression (**Figure 2B**) but when the *S. schleiferi* supernatants were added to the strain expressing the constitutively active AgrC (RN10829 Const.) we observed no difference in RNAIII expression (**Figure 2C**). These results suggest that the effect is AgrC mediated.

**FIGURE 3:**
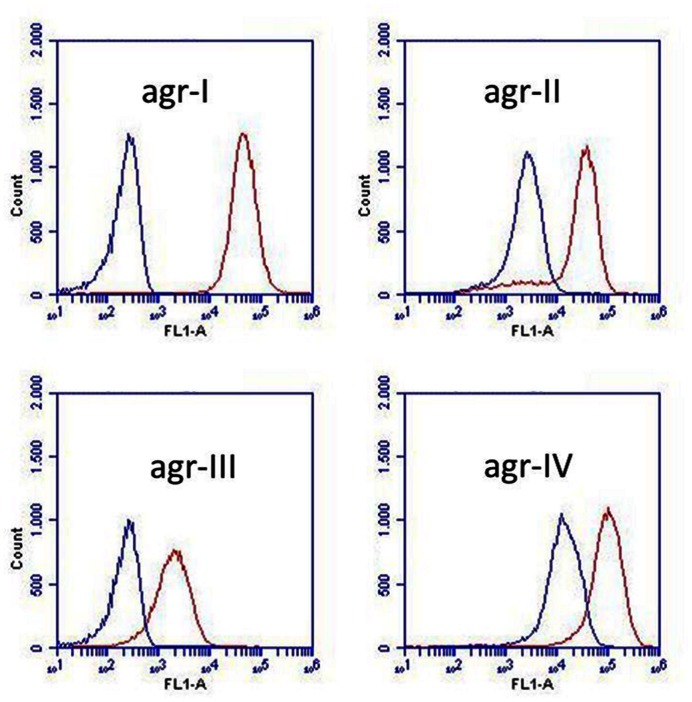
***Staphylococcus schleiferi* supernatant affects the activity of all four *S. aureus agr* types.** Activity of four *S. aureus agr* group reporters (agr-I, agr-II, agr-III, and agr-IV) assessed by P3 expression (P3-*yfp*) measured as YFP accumulation by flow cytometry. Non-fluorescent, exponential phase cells were grown with (blue) or without (red) 20% *S. schleiferi* 2898 supernatant until stationary phase. Depicted fluorescence histograms originate from cells analyzed at the 24 h time point.

Under the presumption that *S. schleiferi* produces AIPs, which cross-inhibit the *agr* system of *S. aureus*, we assessed the specificity with respect to the different *S. aureus agr* types. Hence, we examined expression from a previously described P3-*yfp* reporter construct present in *S. aureus* strains of known *agr* types (Hall et al., 2013) that had been grown in the presence or absence of supernatant from *S. schleiferi* strain 2898. Evidently, the supernatant confered a considerable degree of repression across all *agr* groups of *S. aureus* (**Figure 3**) giving rise to a 10–100 fold reduction in observed fluorescence intensity of all reporters. We note that the *agr* system appears almost completely repressed in the types I and III reporters even at the late time point of the assay (24 h), while the repression of the types II and IV reporters display a delay in the response. This difference may stem from different cross-inhibitory affinities of the suspected AIP molecule of *S. schleiferi* against the different AIP-AgrC receptor pairs in *S. aureus*, or simply reflect a difference in the *agr* activation kinetics and/or auto-fluorescence of the reporters employed.

### *S. schleiferi* Inhibition of *S. aureus agr* is AIP-Mediated

**FIGURE 4:**
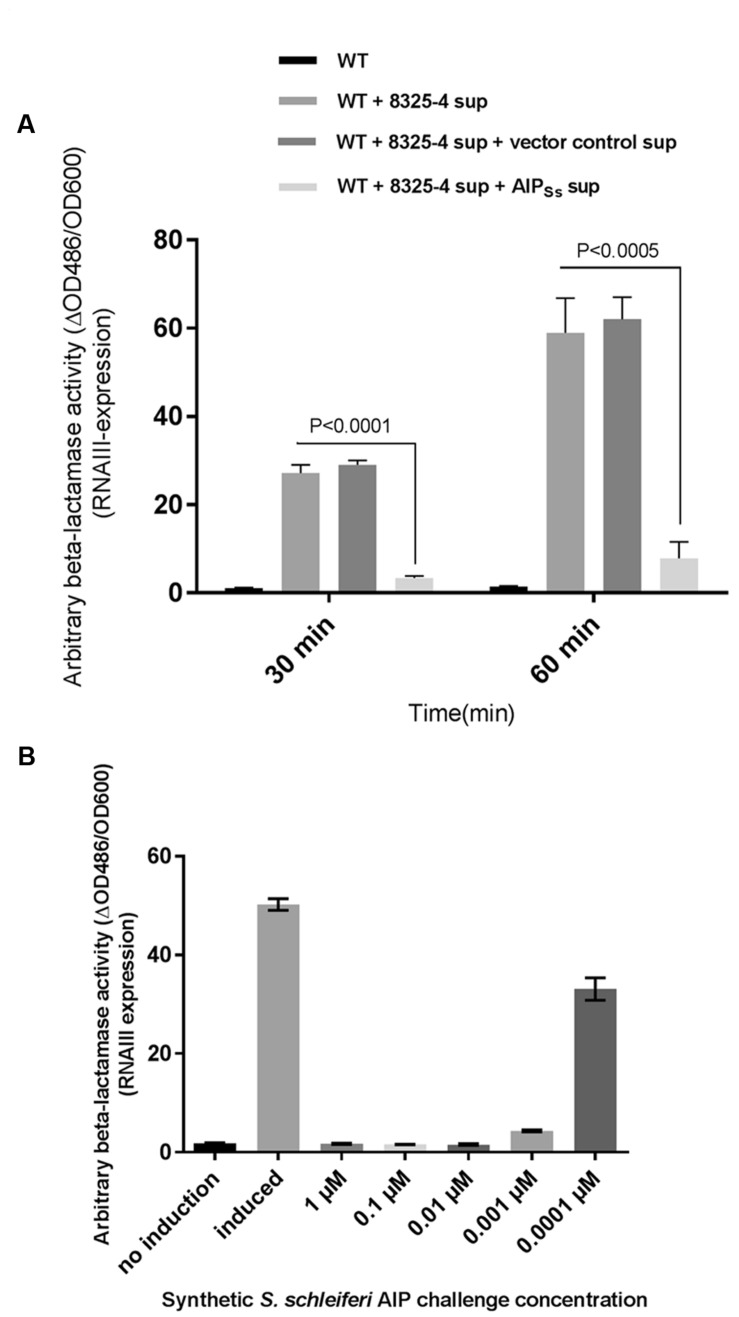
***Staphylococcus schleiferi* AIP interferes with *S. aureus agr*. (A)** RNAIII expression was recorded as β-lactamase expressed from the P3-blaZ reporter fusion in *S. aureus* RN10829(P2-agrA:P3-blaZ)/pagrC-I (WT) with addition of 5% AIP-I supernatant and 20% supernatant from either strain 8325-4Δ*agr*/pRAB12-agrBD_Ss_ (AIP_Ss_) or the control strain 8325-4Δ*agr*/pRAB12-lacZ (vector control) that had been grown and induced (0.2 μg/ml anhydrotetracycline) overnight. Each bar represents the average of three biological replicates and the error bars represent the standard deviation. (**B**) P3-blaZ expression recorded from *S. aureus* RN10829(P2-agrA:P3-blaZ)/pagrC-I (WT) when the inducing AIP-I containing supernatant (10%) is challenged for 45 min with different concentrations of synthetic *S. schleiferi* AIP at indicated concentrations. No induction and AIP-I containing supernatant alone was included as controls. Each bar represents the average of three biological replicates and the error bars represent the standard deviation.

Having demonstrated that *S. schleiferi* supernatant is a potent inhibitor of RNAIII expression in *S. aureus* we investigated the hypothesis that the inhibition was due to the presence of non-native AIP in the supernatant of *S. schleiferi*. *S. aureus* AIP was previously biosynthesized in *S. aureus* by cloning the *agrBD* genes in an otherwise *agr* negative background (Thoendel and Horswill, 2009). Here, after genome sequencing of the *S. schleiferi* strain 2898, we cloned the 2898 *agrBD* genes into the expression vector pRAB12-lacZ (Helle et al., 2011) generating plasmid pRAB12-agrBD_Ss_ and expressed it in the *S. aureus agr* deletion strain 8325-4Δ*agr*. In contrast to the inactive vector control strain the supernatant of the *S. schleiferi*-AIP (AIP_Ss_) producing strain clearly inhibited RNAIII expression as measured by β-lactamase activity from the P3-*blaZ* reporter strain both 30 and 60 min after addition of the supernatant (**Figure 4A**). These results confirm that the AIP produced by *S. schleiferi* is inhibiting *agr* of *S. aureus*. To further support that it is in fact the *S. schleiferi* AIP that is responsible for inhibition of *S. aureus* RNAIII via AgrC agonist activity, we synthesized the proposed *S. schleiferi* AIP and tested the synthetic material in the same P3-*blaZ* reporter strain. Our results show indisputably that the *S. schleiferi* AIP is a potent inhibitor of *S. aureus* RNAIII expression and that it acts antagonistically on the reporter strain at low nanomolar concentrations (**Figure 4B**).

### *S. aureus* agr Repression by *S. schleiferi* is Observed during Niche Competition Both *In vitro* and *In vivo*

**FIGURE 5:**
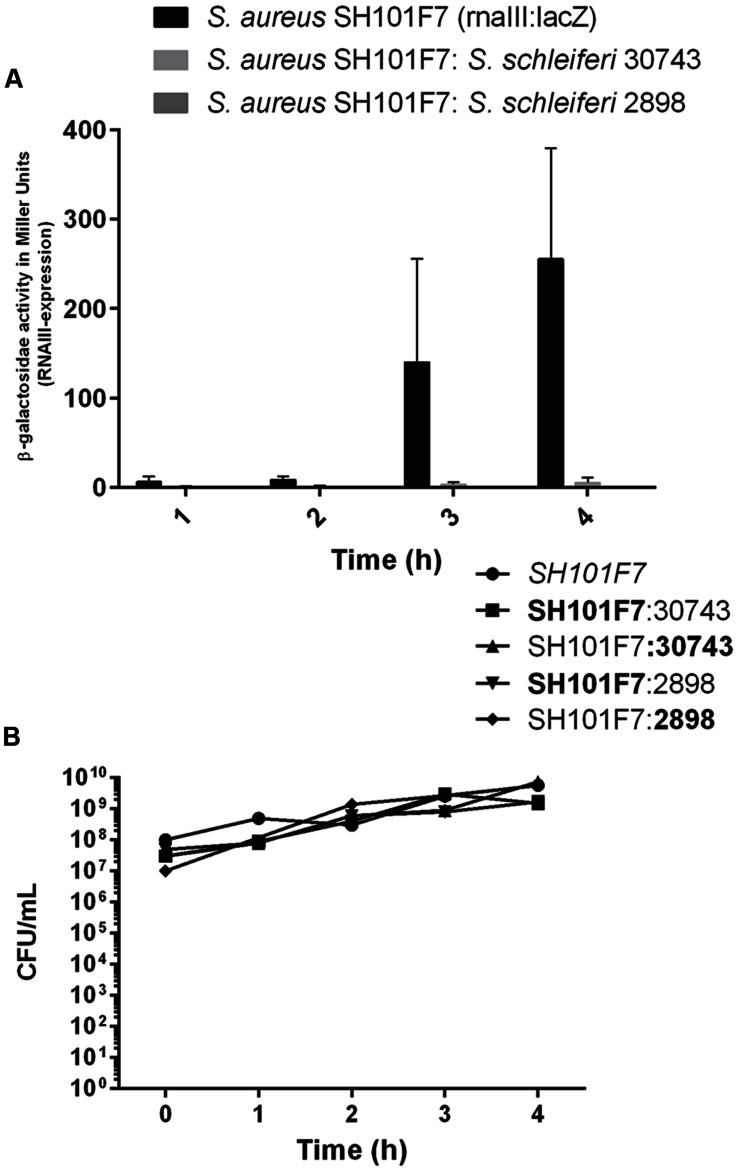
***Staphylococcus schleiferi* inhibits *S. aureus agr* during co-culture without influencing growth. (A)** *S. aureus rnaIII*:*lacZ* reporter strain SH101F7 grown alone or in co-culture (1:1) with *S. schleiferi* strain 30743 or 2898 in TSB. β-galactosidase activity under each condition was determined to monitor *agr* induction. Each bar represents the average of three replicates and the error bars represent the standard error of the mean. **(B)** Growth of the bacterial cultures measured as colony forming units (CFUs)/mL monitored in parallel for each time point. Distinction between the two species in the co-culture was made by plating on TSA substituted with X-gal, resulting in the SH101F7 reporter strain growing as blue colonies, while the *S. schleiferi* growing as white.

To address if the *agr* inhibitory activity of *S. schleiferi* also is expressed when both strains share the same niche environment we co-cultured *S. aureus* SH101F7 *rnaIII::lacZ* reporter strain together with the *S. schleiferi* 2898 or 30743 strains at a 1:1 ratio in TSB. After 4 h where bacterial growth had reached stationary phase and the *agr* quorum sensing system in *S. aureus* is normally fully activated, we observed almost complete inhibition of *S. aureus* RNAIII expression in cells co-cultured with either of the *S. schleiferi* strains (**Figure 5A**). Within the time frame of the experiment, the growth proportion of *S. aureus* to *S. schleiferi* cells remained essentially unchanged suggesting that neither strain exert a growth inhibitory effect toward each other (**Figure 5B**). Thus, during co-culture the presence of *S. schleiferi* efficiently represses the *S. aureus agr* quorum sensing system.

**FIGURE 6:**
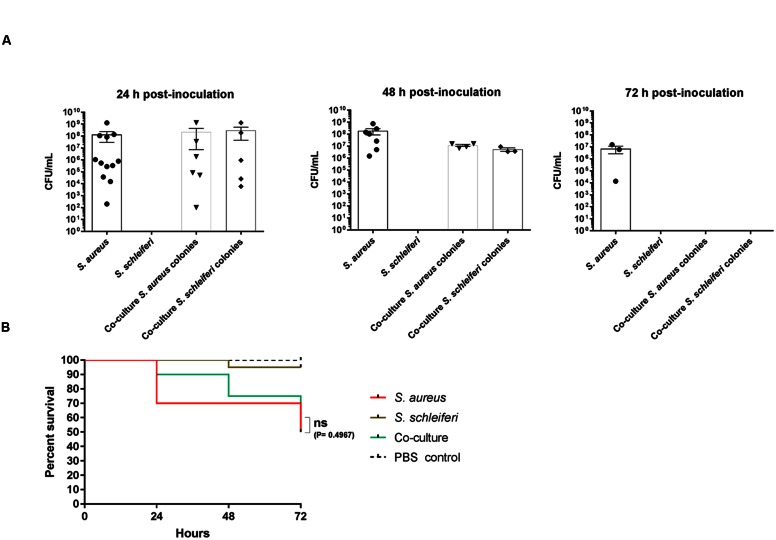
**Dual presence of *S. schleiferi* and *S. aureus* influence colonization capabilities in a *Galleria mellonella* infection model.** Fifth-instar *G. mellonella* larvae were inoculated with a total of 2 × 10^7^ CFU/mL of *S. aureus* (SH101F7), *S. schleiferi* (2898) or a co-culture of the two strains (to a combined final CFU/mL of 2 × 10^7^) and split into groups for CFU counting (35 per group) and survival benefit observation (20 per group). **(A)** At 24, 48, and 72 h post-inoculation, the hemolymph of the larvae was collected for CFU determination. Colonies were counted after O/N incubation at 37°C on TSA containing erythromycin and X-gal, as both strains are erythromycin resistant. **(B)** Survival of the larvae was monitored at the same time points as hemolymph collection. This experiment was repeated three times with similar results.

Given the obvious interaction between the two bacterial species during co-culture in laboratory medium we investigated if a similar phenomenon is observed in *G. mellonella* wax moth larvae, a model previously reported for studies of *S. aureus* virulence (Desbois and Coote, 2011). Applying the same growth conditions as for the *in vitro* co-culture experiment we inoculated 2 × 10^7^ colony forming units (CFUs) into the prolegs of fifth-instar larvae and followed the CFU counts and larval survival over a period of 3 days. When single species were inoculated, *S. aureus* was recovered at 1 × 10^8^ cells 24 h post-inoculation and only declined slightly at 72 h. We were unable to detect any *S. schleiferi* in the larval hemolymph at any of the time points (**Figure 6A**), where survival of this group was comparable to the PBS control larvae group (**Figure 6B**). In contrast, when *S. schleiferi* was inoculated together with *S. aureus* both species were present in equal numbers at 24 h, followed by a decline at 48 h and complete eradication of both species after 72 h (**Figure 6A**). The presence of *S. schleiferi* 24 and 48 h after inoculation with *S. aureus* suggests that factors produced by *S. aureus* allow maintenance of both species in the larva, while inversely the clearance of both species at 72 h may be suggestive that there is a *S. schleiferi*-mediated repression of *S. aureus* virulence factors via *agr* down-regulation, and that this repression allows the larvae to eradicate the combined staphylococcal population. Despite the clearance of both species at 72 h, the co-culture did not offer a significant larval survival benefit over the single *S. aureus* inoculant group (**Figure 6B**). Nevertheless, our data show that in the presence of *S. aureus*, *S. schleiferi* is maintained for an extended period of time in the larvae and that ultimately their presence leads to eradication of both species.

### AIP Homology in Staphylococci

**FIGURE 7:**
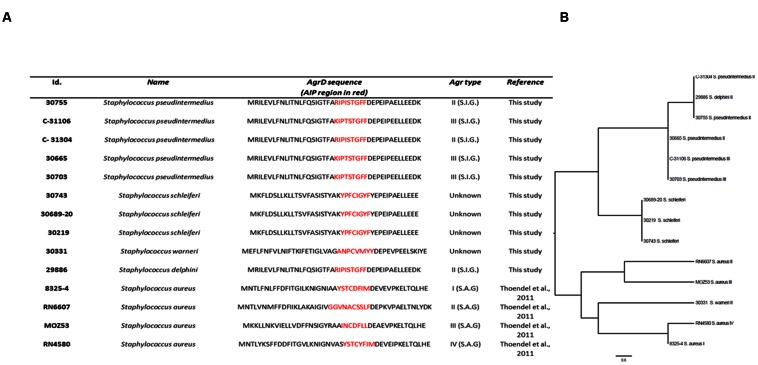
***agrD* homology of selected Staphylococci.** Isolates were analyzed for *agrD* homology by Illumina sequencing; **(A)** Table showing AIP-alignment. The AIP region is highlighted in red. S.I.G.: *Staphylococcus intermedius* Group; S.A.G.: *S. aureus* Group; Strains 8325-4, RN6607, MOZ53, and RN4580 were included as *S. aureus* AIP sequence references. **(B)** Phylogenetic analysis based on a Muscle alignment of the AIP sequences with midpoint rooting. Scale bar indicate substitutions per site.

The AIP amino acid composition, positioning and properties have been reported to play an important role in the type of AgrC interaction (Mayville et al., 1999; Lyon et al., 2000; Wright et al., 2004; Tal-Gan et al., 2013a). Accordingly, we analyzed sequence homology of AIPs from a selection of staphylococci for which supernatant activity ranged from no effect to severe inhibition of *S. aureus agr* (**Figure 7A**). Alignment of AIP sequences between different staphylococcal species revealed less than 30–40% amino acid conservation. Within the same species, the AIPs were highly conserved; a phenomenon that has also been observed for already reported staphylococcal AIP sequences (Dufour et al., 2002; Thoendel and Horswill, 2009). For our sequenced *S. schleiferi* AIPs there was 100% conservation and we noted that the most highly conserved amino acids between species were those with specific properties needed for correct interaction or folding of AIP. For example, the C-terminal amino acid of most of the ascertained AIP sequences is tyrosine or phenylalanine which provides the hydrophobicity essential for binding of the AIP to a putative hydrophobic pocket in the AgrC receptor, but is not required for the antagonistic or the activation activity (Wright et al., 2004). Also they retained the central cysteine necessary for AIP cyclization (Thoendel and Horswill, 2009). Interestingly, despite their strong inhibitory activity toward *S. aureus agr*, neither *S. schleiferi* nor *Staphylococcus delphini* AIP contain an alanine residue, which has been reported to play a role in non-native AIP-AgrC binding antagonism (Lyon et al., 2000; Tal-Gan et al., 2013a). Inferred relationships of the various AIPs revealed that they clustered according to species and for the most part also according to AIP group (**Figure 7B**). The differences in amino acid conservation and the difficulties in locating specific amino acid sequences responsible for non-cognate AIP-AgrC interactions, highlights that spatial arrangement and secondary structure of the AIPs play an important role in the interactions between the non-cognate AIPs and the *S. aureus* Agr-C (Mayville et al., 1999; Lyon et al., 2000; Wright et al., 2004). We speculate, that AgrC is promiscuous in terms of susceptibility to AIP inhibitory structures and this promiscuity allows inhibition by multiple staphylococcal species.

## Discussion

This study looks at the ecological aspects of regulatory cross-talk and niche sharing between staphylococci as mediated via the *agr* quorum sensing system, and delves into the possible effects this cross-talk may have on aspects of virulence and colonization for the human pathogen *Staphylococcus aureus*.

Cell to cell communication via quorum sensing is very common amongst bacteria and promotes both inter and intra-species interactions (LaSarre and Federle, 2013). The *agr* QS system of *S. aureus* is especially sensitive to pheromones produced by *S. aureus* strains of other *agr* groups (Thoendel and Horswill, 2009; Thoendel et al., 2011) or produced by other staphyloccocal species, namely *S. epidermidis* (Otto et al., 2001), and has even displayed sensitivity to compounds produced by other microorganisms of unrelated niche environments such as the marine *Photobacterium halotolerans*-derived QS inhibitor solonamide B (Nielsen et al., 2010, 2014). Despite this well-documented *agr* cross-inhibition between the different *S. aureus agr* groups and between *S. aureus* and other staphylococcal species, we aimed to explore further the communication between *S. aureus* and staphylococci with little niche overlap, and what repercussions this communication might have in terms of virulence adaptation. While our findings corroborate those of others with regards to staphylococcal cross-talk via *agr* (Otto et al., 2001; Lina et al., 2003; Thoendel et al., 2011), we also show extensive AIP-mediated cross-talk between previously untested staphylococci and *S. aureus*, with 82% of the previously untested staphylococci exerting ability to interfere with *agr* of *S. aureus*. Importantly, we show that staphylococci from varying environmental niches not only have the capacity to interfere with *S. aureus agr*, but even influence virulence and colonization capabilities of *S. aureus* when forced to share a niche, as was shown in the co-infection of *G. mellonella* with *S. schleiferi* and *S. aureus*. Cross-communication involving *agr* interference has previously been observed as a result of co-habitual competition within the same ecological niche, and we speculate that this also is a reason for the observed interference in our study. A few known examples are the interactions between *S. aureus* and *S. epidermidis* on human skin and between *S. aureus* and *Pseudomonas aeruginosa* in the lungs of patients suffering from cystic fibrosis (Otto et al., 2001; Qazi et al., 2006). In the first example, *S. epidermidis* AIP pheromone is capable of inhibiting *agr* activity of *S. aureus* groups I, II and III, but not that of group IV, which interestingly is the only *S. aureus* AIP capable of inhibiting *S. epidermidis agr* activity. It has been suggested that this cross-talk is one of the reasons why *S. epidermidis* predominates over *S. aureus* on human skin (Otto et al., 2001). In the second example, the cross-talk between *P. aeruginosa* and *S. aureus* is a case of cross-communication between two unrelated microorganisms. It has been shown that *N*-acylhomoserine lactone 3-oxo-C_12_-HSL produced by *Pseudomonas* is capable of inhibiting *S. aureus* growth, and that this compound at sub-inhibitory concentrations interferes with *S. aureus agr* and *sarA* expression in a manner involving cytoplasmic membrane saturation (Qazi et al., 2006). In cystic fibrosis patients, the interaction between them in addition to host factors tends to favor the predominance of *S. aureus* colonization in young patients and *P. aeruginosa* in adult patients, though co-isolation of both organisms is still found in 50% of adult CF patients (Qazi et al., 2006). The co-existence of these two organisms in CF patients suggests an evolutionary role of cross-talk on influencing virulence (Qazi et al., 2006; Pastar et al., 2013). Our data on the effect of *S. schleiferi* on *S. aureus* virulence in the *G. mellonella* host suggests that this influence on virulence via QS cross-talk may be a common mechanism, and offers a good model for further exploration of this evolutionary role and a possible tool to study virulence adaptation. One limitation of our study is the lack of isogenic *S. schleiferi agr* mutants, which would allow us to firmly establish *agr* interference as the main cause of the characteristic co-colonization pattern observed in the *in vivo* model. At present, we cannot exclude the involvement of other factors during niche-sharing. Nevertheless, it still remains baﬄing that so much cross-talk or interference via QS is observed between *S. aureus* and distant staphylococci. Although an attempt to understand this was made by sequencing several AIP-encoding genes, the exact mechanisms of this AgrC promiscuity that allows susceptibility to non-cognate AIP are yet to be determined. Further studies that apply a systematic analysis of the *S. schleiferi* AIP structure and characterization of key structure-activity-relationship functions necessary for *S. aureus* AgrC activation/modulation or inhibition are needed. Following in the footsteps of the pioneering work described by Blackwell colleagues on *S. aureus* and *S. epidermidis* AgrC modulation (Tal-Gan et al., 2013a,b, 2016; Yang et al., 2016), would certainly shed light on the key features responsible for AgrC promiscuity.

Apart from the insight into ecological aspects of regulatory cross-talk and niche sharing, our findings could also be of potential therapeutic interest. The observation that even at nanomolar concentrations the *S. schleiferi* AIP is a potent inhibitor of *S. aureus agr* and is active across all *agr* specificity groups I to IV is exciting, offers a potential new avenue for exploring staphylococci as sources for quorum sensing inhibitors that can be used to target *S. aureus agr*-related infections. As we are now faced with the ever increasing burden of antimicrobial resistance, alternative therapeutic avenues are being explored to treat infections with antibiotic resistant pathogens, such as methicillin-resistant *S. aureus* (MRSA) (Boucher et al., 2009). One such alternative approach is anti-virulence therapy, that targets virulence rather than viability of the pathogen (Rasko and Sperandio, 2010). For MRSA, QS inhibition via the application of compounds that target the *agr* system has been shown to be a promising anti-virulence approach (Nielsen et al., 2014; Sully et al., 2014; Baldry et al., 2016). The potent *S. aureus agr*-inhibitory activity of *S. schleiferi* AIP fits perfectly within the scope of anti-virulence therapy targeting *S. aureus*, and warrants further *in vivo* investigations to address the true therapeutic potential of staphylococcal derived anti-virulence candidates targeting *S. aureus*. Furthermore, the significance of our findings are very much in line with the very rescent discovery that human nasal commensal staphylococci are also potential sources of novel antibiotics to combat MRSA (Zipperer et al., 2016).

## Conclusion

We show that there is extensive cross-talk between *S. aureus* and other staphylococcal species mediated via the *agr* quorum sensing system. We have (i) identified several staphylococcal species with the ability of cross-interfering with *S. aureus agr*, (ii) identified novel AIP sequences, some of which exhibiting broad antagonistic activity on *S. aureus agr*, and (iii) provided a method for expressing foreign AIPs in *S. aureus*. As staphylococcal species are often found in the same habitat, our results suggest that the *agr* quorum sensing system is important for inter-species communication, that it may play a crucial role in determining the local population structure, and that this communication may influence *S. aureus* virulence. Lastly, we suggest that AIPs from other staphylococci have the potential to be exploited as anti-virulence compounds targeting *S. aureus* infections.

## Author Contributions

MB, MSB, PD, CO, and HI planned the experiments. JC, MB, and MSB performed the microbiological work. BG and PG performed chemical synthesis. MS did the bioinformatic analysis. PA supervised the bioinformatic analysis and PD and HI the microbiological work. CO supervised the chemical research. JC, MB, MSB, PA, PD, CO, and HI contributed to the writing of the manuscript.

## Conflict of Interest Statement

The authors declare that theresearch was conducted in the absence of any commercial or financial relationships that could be construed as a potential conflict of interest.
