# Corrigendum: Cross-Talk between *Staphylococcus aureus* and Other Staphylococcal Species via the *agr* Quorum Sensing System

**DOI:** 10.3389/fmicb.2017.01075

**Published:** 2017-06-08

**Authors:** Jaime Canovas, Mara Baldry, Martin S. Bojer, Paal S. Andersen, Piotr K. Grzeskowiak, Marc Stegger, Peter Damborg, Christian A. Olsen, Hanne Ingmer

**Affiliations:** ^1^Department of Veterinary Disease Biology, Faculty of Health and Medical Sciences, University of CopenhagenFrederiksberg, Denmark; ^2^Department of Microbiology and Infection Control, Statens Serum InstitutCopenhagen, Denmark; ^3^Center for Biopharmaceuticals and Department of Drug Design and Pharmacology, Faculty of Health and Medical Sciences, University of CopenhagenCopenhagen, Denmark

**Keywords:** *Staphylococcus aureus*, *Staphylococcus schleiferi*, quorum sensing, *agr*, quorum sensing inhibition, auto-inducing peptide, cross-talk, anti-virulence therapy

It has come to our attention that in Figure 4B of the original article, we stated that the *S. schleferi* autoinducing peptide has the sequence YPFCIAYF. This peptide was synthesized, tested and found to have inhibitory activity. However, the correct AIP sequence is YPFCIGYF. We have now synthesized this peptide with the protocol stated below, which differs from the one published, and have tested the correct peptide for activity. As demonstrated below, we find that the new peptide has strong agr inhibitory activity, as expected, and, therefore, the conclusions of the paper remain the same.

**Figure 4:**
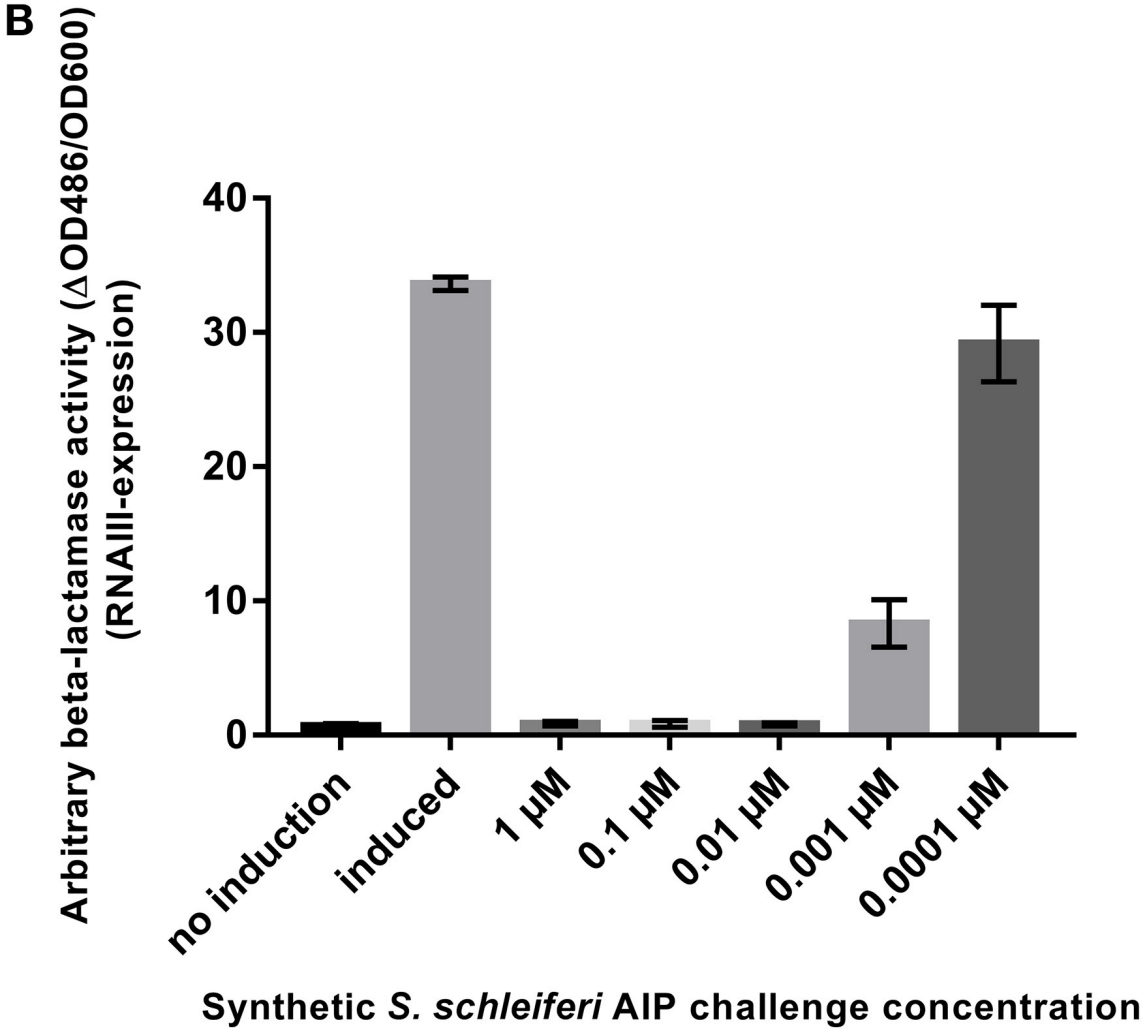
*S. schleiferi* AIP interferes with *S. aureus agr*. **(B)** P3-blaZ expression recorded from *S. aureus* RN10829(P2-agrA:P3-blaZ)/pagrC-I (WT) when the inducing AIP-I containing supernatant (10%) is challenged for 45 min with different concentrations of synthetic *S. schleiferi* AIP at indicated concentrations. No induction and AIP-I containing supernatant alone were included as controls. Each bar represents the average of 3 biological replicates and the error bars represent the standard deviation.

## Materials and methods

### Chemical synthesis of *S. schleiferi* AIP

The *S. schleiferi* AIP was synthesized applying a strategy based on the C-terminal peptide *N*-acyl-benzimidazolinone (Nbz) derivative (Blanco-Canosa and Dawson, 2008), which was previously reported by Blackwell and coworkers (Tal-Gan et al., 2016) for AIP syntheses. Briefly, the linear peptide (20.0 μmol) was synthesized on 3-(Fmoc-amino)-4-aminobenzoyl aminomethyl polystyrene resin (Dawson Dbz AM resin; 0.49 mmol/g, Merck) by automated Fmoc solid-phase peptide synthesis (SPPS) as described in the Materials and Methods of the manuscript. The peptide (5.00 μmol) was then cyclized by Nbz formation, TFA-mediated cleavage from the resin, and stirring in aqueous guanidinium chloride (6 M in 0.1 M phosphate buffer, pH = 6.8)–MeCN (6:4) for 2 h at 50°C (Tal-Gan et al., 2016). Cyclization was followed by purification as described in our manuscript to give the title compound as a white fluffy solid (1.9 mg, 28%).

Purity: >98% as determined by UPLC–MS analysis at 230 nm.

MS: m/z calcd for C_52_H_62_N_8_O_10_S 991.4. ESI-MS found 991.4 [M+H^+^]. MALDI-TOF MS found 991.5 [M+H^+^].

### β-lactamase assay and inhibitory concentration (IC_50_)

The method used is described by Nielsen et al. (2014). Briefly, the RN10829 (P2-agrA:P3-blaZ)/pagrC-I (WT) and RN10829(P2-agrA:P3-blaZ)/pagrC-I-R23H (AgrC const.) reporter strains were grown to an OD_600_ of 0.4–0.5 where a 1/10 volume of AIP-I containing supernatant (obtained from strain 8325-4) and 1/10 *S. schleiferi* supernatants were added to the reporter strain culture. In assays using heterologously expressed AIP_Ss_ 1/20 volume of AIP-I containing supernatant was challenged with 1/5 volume supernatant from expression cultures. Samples obtained at 30 min time intervals after addition of test solutions were analyzed for β-lactamase activity by nitrocefin conversion. The IC_50_ of the selected *S. schleiferi* supernatants was also tested using the β-lactamase assay, where a 1/10 volume (0.5 mL) of supernatant was added to the total volume of 5 mL of the reporter strain culture (RN10829-WT) representing the undiluted supernatant (100%). Then, 80, 60, 40, 20, 10, 5, 2.5 and 2% of the initial volume of the selected supernatant was added to obtain the IC_50_ curve. Statistical analysis was performed using the Student's *t*-test (2-tailed).

## Results

### *S. schleiferi* inhibition of *S. aureus agr* is AIP-mediated

To support that the *S. schleiferi* AIP that is responsible for inhibition of *S. aureus* RNAIII via AgrC agonist activity, we synthesized the *S. schleiferi* AIP with the sequence YPFCIGYF and tested the synthetic compound in the P3-*blaZ* reporter strain. Our results in this amended Figure 4B show that the *S. schleiferi* AIP is a potent inhibitor of *S. aureus* RNAIII expression and that it acts antagonistically on the reporter strain at low nanomolar concentrations.

### Conflict of interest statement

The authors declare that the research was conducted in the absence of any commercial or financial relationships that could be construed as a potential conflict of interest.
