# Corrigendum: Cross-Talk between *Staphylococcus aureus* and Other Staphylococcal Species via the *agr* Quorum Sensing System

**DOI:** 10.3389/fmicb.2017.01949

**Published:** 2017-10-05

**Authors:** Jaime Canovas, Mara Baldry, Martin S. Bojer, Paal S. Andersen, Bengt H. Gless, Piotr K. Grzeskowiak, Marc Stegger, Peter Damborg, Christian A. Olsen, Hanne Ingmer

**Affiliations:** ^1^Department of Veterinary Disease Biology, Faculty of Health and Medical Sciences, University of Copenhagen, Frederiksberg, Denmark; ^2^Department of Microbiology and Infection Control, Statens Serum Institut, Copenhagen, Denmark; ^3^Center for Biopharmaceuticals and Department of Drug Design and Pharmacology, Faculty of Health and Medical Sciences, University of Copenhagen, Copenhagen, Denmark

**Keywords:** *Staphylococcus aureus*, *Staphylococcus schleiferi*, quorum sensing, *agr*, quorum sensing inhibition, auto-inducing peptide, cross-talk, anti-virulence therapy

Bengt H. Gless was not included as an author in the published article. The authors apologize for this error and state that this does not change the scientific conclusions of the article in any way.

The original article has been updated.

## Author contributions

MB, MSB, PD, CO, and HI planned the experiments. JC, MB, and MSB performed the microbiological work. BG and PG performed chemical synthesis. MS did the bioinformatic analysis. PA supervised the bioinformatic analysis and PD and HI the microbiological work. CO supervised the chemical research. JC, MB, MSB, PA, PD, CO, and HI contributed to the writing of the manuscript.

### Conflict of interest statement

The authors declare that the research was conducted in the absence of any commercial or financial relationships that could be construed as a potential conflict of interest.

